# Work and Hobbies

**Published:** 1911-04-29

**Authors:** 


### WORK AND HOBBIES.

To the majority of men some other interest in life than their work is indispensable. It is only a few of us who can attain to old age and keep in harness to the end: and they are men whose work, like that of Professor Metchnikoff, embraces very broad fields of medical science. To most there comes a time when they feel past work, or from whom a large part of their work is taken away when they reach some stated age, as happens in the case of the Army man or the hospital physician. If during the active period of their existence their mind has never turned away from work, they have laid up for themselves a miserable time for the closing period of their lives.

To many medical men the sports of youth remain hobbies to the end of life; when they can no longer plaf them, they read of them and go to see them. Others, again, interest themselves in some public work and become justices of the peace or guardians. Art interests a very large number of the profession: many are keen collectors, on a small scale, of china or of prints, while others gather old pieces of furniture. The medical training has some bearing on this, for the doctor has studied the human frame more closely than any other man, and so he is almost bound to be interested in portraiture and figure painting. He starts with a basis for criticism and this leads him naturally on to other forms of art. The late Sir Seymour Haden was originally a busy practitioner, who taught himself etching in his spare moments in Kensington Gardens. Others, again, take up some science as their hobby; one of the first authorities on beetles at the present time is medical officer to a large public school. Frequently this hobby has some close relation to the doctor's work. Some of the most valuable skulls in the anthropological section of the Royal College of Surgeons' Museum formed originally the collection of a busy practitioner. Sir James Paget spent his holidays in studying the pathology of fruits and plants, a recreation that resulted in his address on Elemental Pathology to the British Medical Association in 1880. It is when a hobby is thus combined with work that the best results come of it.

Mr. Bland-Sutton is a very busy surgeon who is at the same time a pathologist, and who brings into his pathology a large knowledge of comparative anatomy. Twenty years ago this combination of work and hobby resulted in the Erasmus-Wilson lectures on the value of comparative pathology to philosophical surgery, and these were followed by his book on "Tumours, Innocent and Malignant," of which the fifth edition is before us. In this latest edition of a deservedly popular book, wide clinical experience is joined to wide knowledge, and the realms of the history of medicine and of ancient mythology, the literatures of many languages, combine, with his extensive knowledge of comparative anatomy, to make up a work that will be referred to many years after the author has passed away. The general practitioner will find in the perusal of this work on a subject of such paramount importance not only an intellectual stimulant, but a mass of information both instructive and interesting, of a nature such as no other work can boast. There is, as we are quite aware, some prejudice against purely pathological works: the preference is all for books which deal with clinical medicine or surgery, and comparative histology and morbid anatomy find few readers outside the ranks of students preparing for examinations, and specialists. One reason of this prejudice is, perhaps, the horrible manner in which most English books on pathology have been written: Wilks and Moxon is long out of date and no newer work has a tithe of its charm and finish. Mr. Bland-Sutton's book, however, has a charm of its own, and owes much of its attractiveness to the fact that it is a striking example of what may be turned out by a surgeon who is as active in his professional work as he is in cultivating his hobbies. For that reason alone it should appeal especially to the practitioner.

